# 16S rRNA Amplicon Sequencing of Microbial Biofilms from Marsberg Copper Mine, Germany

**DOI:** 10.1128/MRA.01315-20

**Published:** 2021-02-04

**Authors:** Sania Arif, Elias Schliekmann, Michael Hoppert

**Affiliations:** a Department of General Microbiology, Institute of Microbiology and Genetics, Georg-August-Universität, Göttingen, Germany; Indiana University, Bloomington

## Abstract

The 16S rRNA amplicons from biofilms inhabiting rocks near various water bodies of Marsberg Copper Mine (Rhenish Massif, Germany) reveal the diversity of their microbial communities. The abundance of *Chloroflexi* and *Cyanobacteria* taxa in the biofilms near leachate streams indicated the selective enrichment of *Ktedonobacteria* and *Oxyphotobacteria* members.

## ANNOUNCEMENT

Marsberg Kilianstollen Copper Mine (Hochsauerland District, North Rhine-Westphalia, Germany) is known for its copper-rich Lower Carboniferous alum shales and lydites as well as copper remineralizations; previous investigations revealed the geochemistry of heavy metals, copper in particular, in (acidic) sulfidic mine waters (1). In this study, selective enrichment of putatively heavy metal-tolerant microbial communities under copper stress is revealed by 16S rRNA amplicon sequencing of biofilms, sampled on rock surfaces near a spring water stream, on mine unconsolidated rock samples, and in acidic leachate stream sediment.

In February 2018, at Marsberg Copper Mine (51.453502°N, 8.861703°E), biofilms growing near or in diverse mine drainages, ranging from fresh stream water to copper-rich acidic leachate, were aseptically collected and stored at −20°C. The biofilm samples MB1 through MB6 were collected from colonized rocks of the adit wall, within 25 to 35 cm of an acidic leachate stream, MBS18 was taken directly from the acidic leachate outflow stream sediment, samples MBS1 through MBS4 were collected from colonized rocks near the fresh spring water stream, and the biofilms from unconsolidated mine rocks and a wooden plank used for timbering were labeled as MBS11, MBS13, and MBS10 (Table 1). The microbial genomic DNA extraction was carried out by following the standard procedures of the PowerSoil DNA isolation kit (Qiagen, Venlo, the Netherlands). The 16S rRNA V3 to V4 hypervariable regions were amplified with the MiSeq 16S amplicon PCR forward primer 5′-GTCGGCAGCGTCAGATGTGTATAAGAGACAGCCTACGGGNGGCWGCAG-3′ and reverse primer 5′-GTCTCGTGGGCTCGGAGATGTGTATAAGAGACAGGACTACHVGGGTATCTAATC-3′) (2). The amplicons were purified from primers and PCR reagents via the GeneRead size selection kit (Qiagen), according to the manufacturer’s instructions. The purified amplicons were sequenced using the MiSeq platform via the 2 × 300-bp paired-end method and a MiSeq v3 reagent kit (Illumina). FASTQ reads were submitted to the automated pipeline for metagenomic analysis using MetaAmp (http://ebg.ucalgary.ca/metaamp/) with optimized parameters. The minimum length of overlap was set to 24 bp, and the maximum number of mismatches in the overlap region and primer sequences was adjusted to 4 (3). The pipeline employed UPARSE to perform operational taxonomic unit (OTU) clustering. Each OTU was taxonomically assigned at 97% sequence identity against the SILVA v138 database (https://mothur.org/wiki/silva_reference_files/) by Mothur (4, 5).

**TABLE 1:** Sampling sites and sample information^*a*^

Sampling site	Sample name	SRA accession no.	Sample type
Spring water stream	1MBS	SRR12876555	Colonized rock sample
	2MBS	SRR12876554	
	3MBS	SRR12876549	Whitish terrestrial biofilm
	4MBS	SRR12876550	
Zement-Kupferplatte copper precipitation flume drained with acidic orange-colored leachate	MB1	SRR12876544	Colonized rock sample
	MB2	SRR12876543	
	MB3	SRR12876542	Greenish-white terrestrial biofilm
	MB4	SRR12876553	
	MB5	SRR12876552	
	MB6	SRR12876551	
Supporting wooden plank	10MBS	SRR12876548	Soft surface biomass
Mine unconsolidated rock	11MBS	SRR12876547	Whitish terrestrial biofilm
	13MBS	SRR12876546	
Outflow greenish-blue-colored leachate stream	18MBS	SRR12876545	Liquid sample

a BioProject accession number PRJNA670497.

The prevalent phylum taxa were *Actinobacteria* (27%), *Chloroflexi* (17%), *Proteobacteria* (16%), *Cyanobacteria* (12%), *Acidobacteria* (10%), and *Bacteroidetes* (6%) in all samples (MBS1 through MBS4, MB1 through MB6, MBS10, MBS11, MBS13, MBS18). According to the UniFrac weighted algorithm (6), the microbial consortium of the spring water and leachate sample groups were distinct from each other (*P* = 0.034), while the unconsolidated rock group microbiome shared similarity with both the spring water and leachate samples (*P* > 0.097). *Ktedonobacteria*, *Cyanobacteria*, and *Actinobacteria* contributed 26%, 23%, and 16%, respectively, to the biofilm community (MB1 through MB6 and MBS18) growing near the copper acidic leachate stream. The genus-level analysis of all samples identified 10 distinct uncultured *Ktedonobacteria* genera; 80% of these could be classified within the *Ktedonobacteraceae* family. Marsberg Kilianstollen offers a large reservoir of uncultured novel heavy metal-resistant microbial strains, especially belonging to the class *Ktedonobacteria* of *Chloroflexi*.

### Data availability.

The 16S rRNA gene amplicon raw read data sets have been deposited in the NCBI Sequence Read Archive (SRA) under BioProject accession number PRJNA670497, and the individual samples under the accession numbers SRR12876542, SRR12876543, SRR12876544, SRR12876545, SRR12876546, SRR12876547, SRR12876548, SRR12876549, SRR12876550, SRR12876551, SRR12876552, SRR12876553, SRR12876554, and SRR12876555 are publicly published.
